# Damage Identification of Semi-Rigid Joints in Frame Structures Based on Additional Virtual Mass Method

**DOI:** 10.3390/s22176495

**Published:** 2022-08-29

**Authors:** Xinhao An, Qingxia Zhang, Chao Li, Jilin Hou, Yongkang Shi

**Affiliations:** 1 School of Civil Engineering, Dalian University of Technology, Dalian 116023, China; 2 School of Civil Engineering, Dalian Minzu University, Dalian 116650, China; 3 Glodon Company Limited, Shanghai 200093, China

**Keywords:** semi-rigid joint, damage identification, virtual mass, natural frequency

## Abstract

In civil engineering, the joints of structures are complex, and their damage is generally hard to be detected. Due to the insensitivity of structural modal information to local joint damage, this paper presents a method based on additional virtual mass for damage identification of a semi-rigid joint in a frame structure. Firstly, the modeling of a semi-rigid is described. Secondly, the frequency response of the virtual structure is constructed, and the natural frequency of the constructed virtual structure is extracted by the ERA method. By adding multiple values of virtual masses at different positions, the natural frequency information sensitive to joint damage for damage identification is effectively increased. Based on the above theory, qualitative identification of joint damage is proposed to detect the potential damage, and identification of both damage location and its extent is presented, using natural frequency. Improved Orthogonal Matching Pursuit (IOMP) algorithm is employed to improve the accuracy of the natural frequency-based method for damage identification. At last, numerical simulation of a three-story frame is performed to discuss and to verify the effectiveness of the proposed method.

## 1. Introduction

During the past few years, new types of beam-column connectors have emerged and are receiving widespread attention in the field of civil engineering [1]. In practice, damage may occur on the structural member, such as beams and columns. Currently, there are rich methods on the damage identification of a structural member [2]. However, for some prefabricated concrete structures and steel structures, joints are relatively more susceptible to damage. Since joints that connect structural members and transmit load and deformation play a significant role on structural safety and reliability, this study focuses on the identification of joint damage only. In general, the connection of joints is simply assumed to be a completely rigid connection or an articulation, however, joints do not behave in such an extreme manner. Articulated joints will generally have a certain degree of rotational stiffness and, similarly, rigid joints will also exhibit a flexible aspect. It can therefore often be considered as a semi-rigid joint [3]. In concrete structures, many beam-column connections can be seen as semi-rigid joints, such as bolted connections of precast concrete beams [4] and precast simple shear beam-column connections [5]. In steel structures, there are also many joint systems which can be considered as semi-rigid, such as the MERO joint systems, the bolt-ball joints, and the space-truss connectors [6]. Damage to semi-rigid joints is caused by a decrease in the strength of high-strength bolts, or cumulative initial bending of the bar under various loads, etc. [7], which, combined with the concentrated shear forces on the joints’ parts, tend to be more prone to damage. Further, semi-rigid joints exhibit different properties compared to rigid joints, and thus the damage identification is different for rigid joints and semi-rigid joints. This study aims to investigate the damage identification of semi-rigid joints.

For frame structures, joints have a significant impact on their performance [8]. Structural joints are usually complex and hidden, their damage is hard to directly measure with instruments. Therefore, damage identification of semi-rigid joints using a structural vibration response is a practical approach. Damage in the structure may cause a change in local stiffness and then the dynamic response of the damaged structure’s change can be compared with that of the intact structure, which can be used to locate and quantify the damage.

Structural damage identification based on vibration data can be mainly divided into two categories: model-based parametric methods and signal-based non-parametric methods. The model-based methods take into account the changes in structural dynamic characteristics, such as natural frequency [9], modal shape [10], frequency response function [11], and dynamic strain response [12,13], and the dynamic characteristics obtained from the measured structural responses are used to update the finite element model so that the modified model can reflect the actual structural health state. Techniques of model updating can be taken as an optimization problem to minimize the difference between the information obtained from an actual damaged structure and the information obtained from the finite element model of the structure. The optimization algorithms used are mainly divided into two categories: traditional optimization methods and intelligent algorithms. Thereof, the optimization process of the former relies on the gradient of the objective function, and it has the advantage of faster convergence, such as the quasi-Newton method [14], the Gauss–Newton method [15], etc. For the latter, the optimization originates from the imitation of biological behavior laws, having the advantage that the search does not require mathematically rigorous expressions of which the representative algorithms include genetic algorithms [16], particle swarm algorithms [17], cuckoo algorithms [18], etc. Currently one of the most representative methods is based on machine learning [19,20,21,22], including two steps of feature extraction and classification of damage types, which requires predefined damage types for model training. For the signal-based method, such as wavelet analysis [23] and Hilbert–Huang transform [24], it does not need to consider the relation between the vibration signal and the model parameters, and statistical or signal processing techniques is used to extract features directly from the original vibration signal and classify the damage state by comparing it with the intact structure. The signal-based method could usually determine the fact of the defect, but it is not easy to determine the location and strength of the defect present in the tested structure.

So far, many studies have been done on damage identification of semi-rigid joints. Hou et al. [25] considered the damage identification of frame structures with semi-rigid joints and proposed a two-step damage detection identification method using frequency and modal array as the objective function. Bharadwaj et al. [26] varied the frequency and modal shape by a moderate amount and estimated the model damage using a unified particle swarm algorithm. Zhou et al. [27] identified the damage of the structures, which was connected by semi-rigid joints and the nonlinear vibration characteristics of structures was considered. Sharma et al. [28] used a modified one-dimensional convolutional neural network to predict the damage occurrence and its location from the semi-rigid joints. Peral et al. [29] processed the time-domain acceleration signal with continuous wavelet transform (CWT) to obtain a scale map and used a two-dimensional convolutional neural network (CNN) architecture for semi-steel joint damage identification. However, most of the data-based methods require building large training datasets and the trained neural networks are only valid for one model. Then, for a completely new structural model, additional training is again required, which is time-consuming. In addition, the labels of the training data are difficult to cover all combinations of working conditions aimed at the potential damage.

Among the structural vibration characteristics, natural frequency belongs to the most fundamental and available modal parameter, but it is hard to use it purely. For example, low-order frequencies, which can be measured accurately in real time, are very insensitive to local damage. Higher-order frequencies are sensitive to changes in local stiffness but are difficult to be excited and estimated accurately. Cha et al. [30] added a specific mass to the structure to update the model parameters using the orthogonality condition of the system eigenvalues. However, it is usually difficult to add real masses to structures on the required positions. Additional virtual parameters including additional virtual mass, stiffness, damping, etc., can improve the sensitivity of the structure to local damage, and thus the construction of virtual structures by adding virtual parameters is more widely used for structure damage identification. Li et al. [31] used the additional virtual mass method to identify structural damage and the method was verified by experiments of a simply supported beam and a 3D truss structure. Zhang et al. [32] identified the damage of a bridge by adding a much larger virtual mass to the bridge than an ordinary vehicle, which provided enough modal information for damage identification. Thus, this study investigates damage identification via additional mass for semi-rigid joints of frame structures.

This paper is structured as follows. In Section 2, modeling of semi-rigid joints is introduced. In Section 3, the construction of virtual structures with additional virtual mass is briefly described, and damage identification methods are presented for the semi-rigid joints of a frame structure based on additional virtual mass. Section 4 verifies the effectiveness of the proposed methods using numerical simulations.

## 2. Modelling of Semi-Rigid Joint

**Figure 1:**
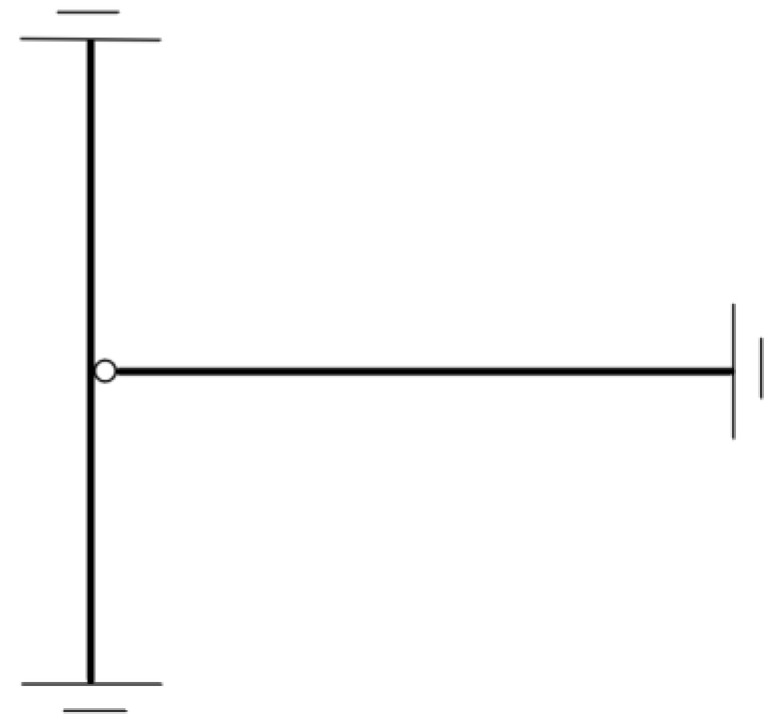
Ideal articulation.

**Figure 2:**
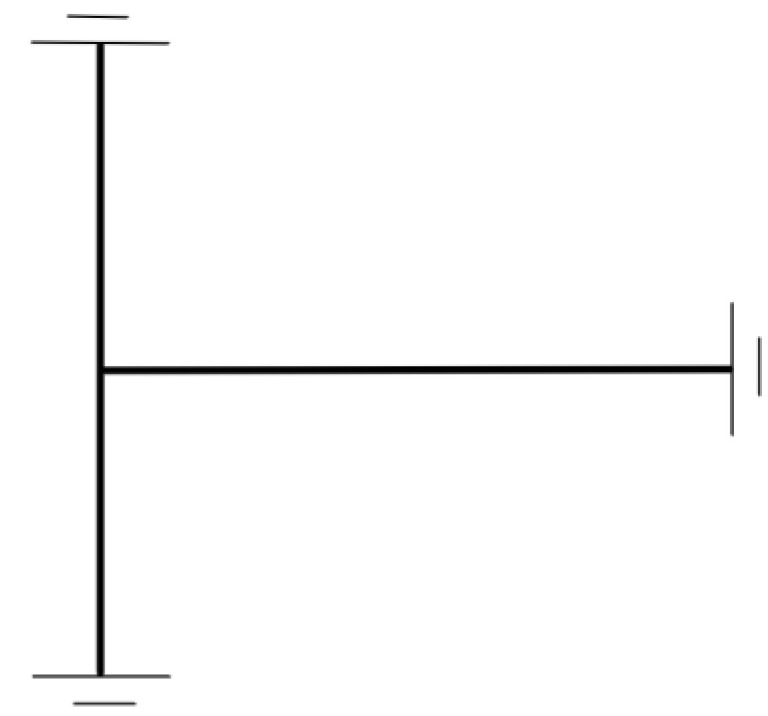
Fixed connection.

**Figure 3:**
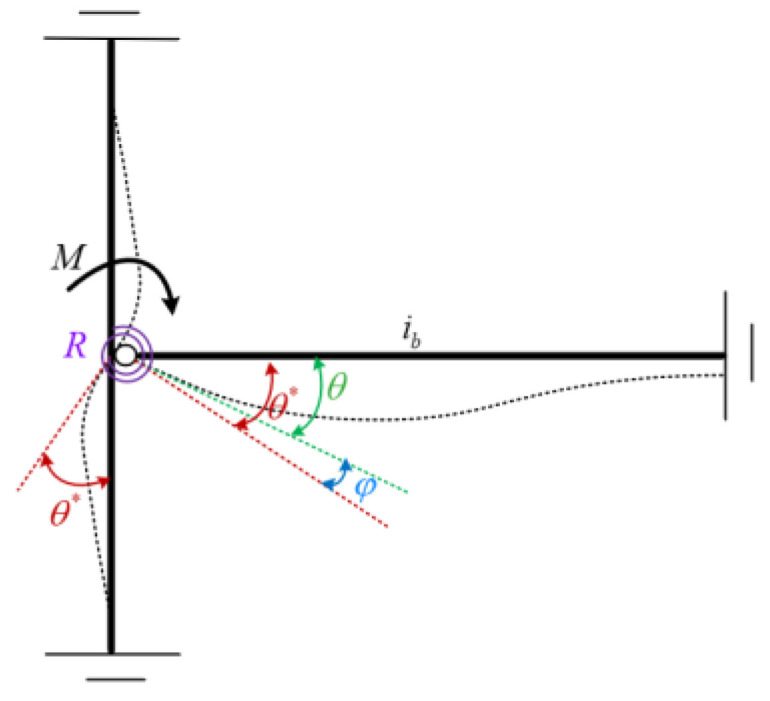
Semi-rigid joint model.

Here, a semi-rigid joint refers to the joint that is between ideal articulation (Figure 1) and fixed connection (Figure 2), and this joint has kind of a rotational stiffness and can withstand part of the bending moment. This article focuses on the study of the semi-rigid beam-column joint of frame structures, and it can be modelled by an elastic rotating spring element, as shown in Figure 3. Denote θ* as the angle of the column end, *θ* is the angle of the beam, and φ is the angle caused by the rotation of the joint spring, which is called the relative rotation of the joint. The relation between angle θ* and angle φ is shown in Equation (1).

(1) $$\varphi =\theta^{*}-\theta$$

Denote *R* as the rotational stiffness of the joint, then φ can also be expressed in terms of the relative rotational stiffness *R* and the bending moment *M*, which is applied on the joint, as shown in Equation (2):

(2) φ=M/R

Assume that the beam line stiffness *i_b_* is evenly distributed along its length. Define *β* as the ratio of the joint stiffness *R* to the beam line stiffness *i_b_*, i.e., *β* = *R*/*i_b_*. In the finite element model of the semi-rigid joint beam element, the semi-rigid joint can be considered based on a reasonable value range of *β* [33], as shown in Table 1.

Generally, in the analysis of structural dynamics the semi-rigid joint is regarded as an independent basic element, which participates in the assembly of the stiffness matrix of the finite element model. Its element stiffness matrix is expressed as shown in Equation (3). To implement semi-rigid joints in the finite element model, the FEM model with ideal articulation (Figure 1) is created and then the stiffness of semi-rigid elements (Equation (3)) are added to the articulated beam ends.

(3) $$K_{es}=R\left[\begin{array}{cc} 1 & -1\\ -1 & 1 \end{array}\right]$$

## 3. Damage Identification Based on Additional Virtual Mass

Here based on Ref. [34], firstly, the construction of virtual structures by adding virtual masses is described, and the sensitivity analysis to the damage factor is also introduced, which is required for determining the value of the additional mass. Then, two methods for damage identification of semi-rigid joints are derived via the virtual structures. Joint damage is simulated by the change of the rotational stiffness.

### 3.1. Construction of Virtual Structure

#### 3.1.1. The Concept of Additional Virtual Mass Methods

**Figure 4:**
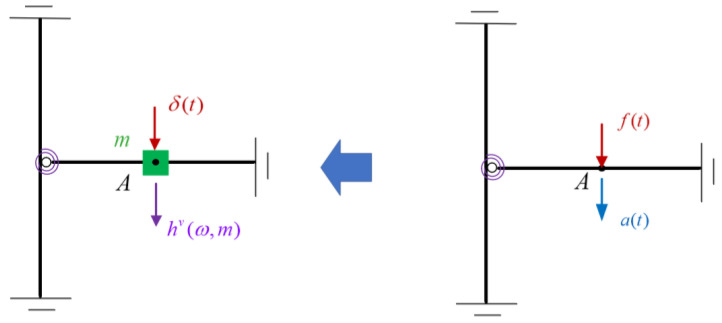
Diagram of the frequency response construction of the virtual structure.

The frequency response construction of the virtual structure is shown in Figure 4. In the left figure, *m* denotes the additional virtual mass added to the virtual structure at point A, *δ*(*t*) denotes the unit impulse excitation applied to the virtual structure at point A, and $h^{v}\left(\omega ,m\right)$ denotes the frequency response of the vertical acceleration response at point A to *δ*(*t*), where the superscript *v* represents the virtual structure. Assuming that *m* is to be added at point A and the frequency response $h^{v}\left(\omega ,m\right)$ of the virtual structure is desired, it is only necessary to apply a vertical excitation *f*(*t*) at point A of the original structure (the right figure), as required by the virtual mass method. Although *f*(*t*) can excite oscillations in different directions and possibly even torsional vibrations, the method requires the measurement of only one acceleration response, i.e., the acceleration response *a*(*t*) at the additional virtual mass position (point A) in the same direction as *f*(*t*). Substituting *f*(*t*) and *a*(*t*) into Equation (4), the frequency response $h^{v}\left(\omega ,m\right)$ of the virtual structure with the added mass *m* can be calculated. With Equation (4), the modal information of the virtual structure can be estimated mathematically by the measured data of the actual damaged structure and the virtual mass values [34].

(4) $$h^{v}\left(\omega ,m\right)=\frac{a\left(\omega \right)}{f\left(\omega \right)+ma\left(\omega \right)}$$

#### 3.1.2. Constructing Multiple Virtual Structures

**Figure 5:**
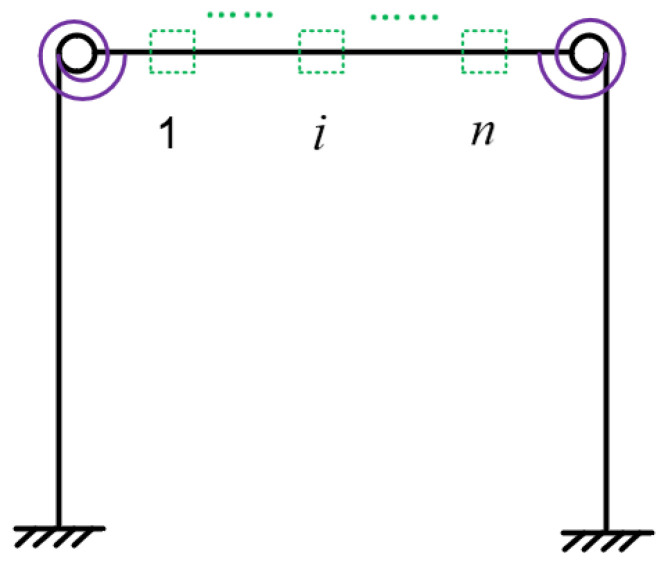
Positions of the additional virtual mass.

In order to obtain enough information for structural damage identification, multiple virtual structures are required to be constructed with regard to the virtual mass added at different positions. As shown in Figure 5, assume that a virtual mass can be added in turn on *n* positions of the structure and take the case of viral mass added on the *i*th position to describe the construction of the virtual structure, i.e., the ith virtual structure. Denote *f_i_*(*ω*) as the excitation applied at the ith position of the actual frame, and let *a_i_*(*ω*) be the acceleration along the same DOF of the excitation *f_i_*(*ω*). For a virtual mass m added on the ith position along the same DOF, the acceleration frequency response of the ith virtual structure $h_{i}^{v}\left(\omega ,m\right)$ can be constructed using Equation (4) via the measured *f_i_*(*ω*) and *a_i_*(*ω*). Using the above method, *n* virtual structures can be constructed by sequentially performing dynamic tests at *n* positions on the actual structure. In addition, since the additional virtual mass values are changeable, the values can be changed to construct different virtual structures. For the same position, multiple values of virtual mass can be added to construct different virtual structures. Assuming that *Q* virtual masses are sequentially attached to *n* locations, it is possible to construct *Q* × *n* virtual structures, which is beneficial to increase the data used for damage identification. The natural frequencies of the constructed virtual structures are different due to their different mass matrices, and in this way the modal information of multiple virtual structures can be constructed arbitrarily for damage identification of actual structure.

#### 3.1.3. Sensitivity-Based Method for Determining the Value of Virtual Mass and Frequency Order

Denote ***μ*** as the damage factor of a semi-rigid damaged joint, which is the scale factor of the stiffness reduction. For ***μ*** = **1**, it means the structure is intact. Assume that there are *n* joints to be identified in the structure, and $\mu ={\left[\mu_{1},\;\mu_{2},\ldots ,\mu_{n}\right]}^{T}$ is a column vector consisting of *n* damage factors. According to the equation of force equilibrium, the generalized eigenvalue equation is established and then the first-order partial derivative is found for it. The formula for the frequency sensitivity of the *k*th joint at the *j*th order frequency to damage factor ***μ*** can be obtained as follows:

(5) $$R_{ji,k}\left(\mu ,m\right)=\frac{\partial \omega_{ji}\left(\mu ,m\right)}{\partial \mu }=\frac{\Psi_{ji}^{T}\left(\mu ,m\right)K_{k}\Psi_{ji}\left(\mu ,m\right)}{2\omega_{ji}\left(\mu ,m\right)}$$

where i is the position of the additional mass, *K_K_* is the stiffness matrix of the *k*th joint, $\omega_{ji}\left(\mu ,m\right)$ is the *j*th order natural frequency when the virtual mass is added on the *i*th position, and $\Psi_{ji}$ is the corresponding modal shape to the *j*th order natural frequency when adding mass on the ith position. For different joints, the value of the additional mass affects its sensitivity to local damage, so a sensitivity analysis is needed to determine the additional mass value. For joint k, when the position i of the additional virtual mass is decided, the sensitivity $R_{ji,k}\left(\mu ,m\right)$ is compared for each order of frequency (j=1,2….) by adjusting the value of *m* as a way to determine the natural frequency order with the greatest sensitivity and the required value of *m*. The selected natural frequency order is most sensitive to damage of joint *k*.

### 3.2. Qualitative Identification of Damaged Joints

Structural joint damage is hard to accurately identify because the whole structure generally is not sensitive enough to local damage. Here a qualitative identification of joint damage is proposed by adding virtual masses at appropriate positions, which affords a fast approach to approximately evaluate the joint condition.

#### 3.2.1. Influence of the Additional Virtual Mass Position

**Figure 6:**
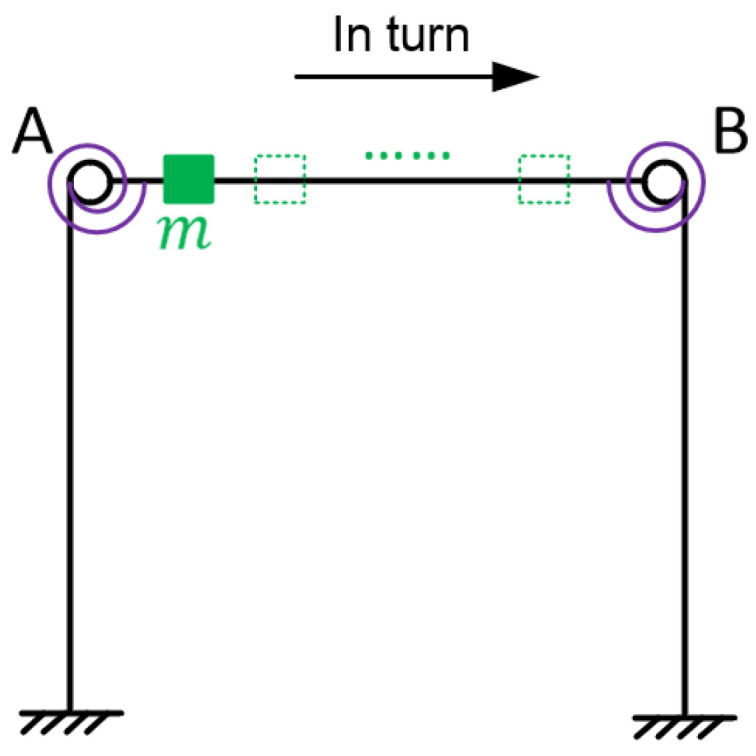
A simple frame structure model.

Take a simple frame structure as an example shown in Figure 6, where joint A and joint B are both semi-rigid joints. Assume four cases which are as follows: no damage, joint A is damaged, joint B is damaged, joint A and B are both damaged.

**Figure 7:**
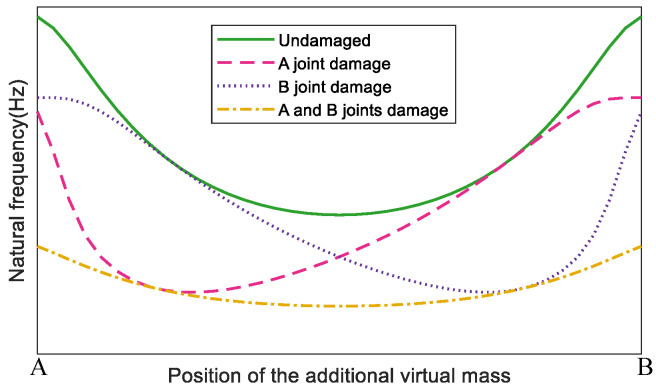
The relation curves of the second order natural frequency of virtual structures with the positions of the added virtual mass in four damage cases.

In each damage case, a virtual mass is added in turn at different positions of the beam from joint A to joint B, and correspondingly the virtual structure is constructed with regard to the virtual mass position. When the value of the additional virtual mass is decided, the sensitivity analysis shows that the most sensitive natural frequency to joint damage for all virtual structures constructed on the basis of this structure is the second order. The sensitivity analysis method will be shown in detail in the numerical simulation section. Take the position of the additional virtual mass as the horizontal coordinate, and the second order natural frequency, for instance, obtained from the corresponding virtual structure as the vertical coordinate, their relation curves in the considered four damage cases are shown in Figure 7.

From Figure 7, it can be seen that in the case where the semi-rigid joints are not damaged, the frequencies of the virtual structure are symmetrically distributed with the position of the additional mass, and the changes are large. In the case of joint A or B being damaged, the frequency decreases to the maximum when the position of the added virtual mass is approximately 1/4 of the beam length to the damaged joint. In the case of joints A and B having the same damage, the change of the structural frequencies is symmetrically distributed and their values do not decrease by much.

In summary, when the position of the additional virtual mass is close to the damaged joint, the change of the natural frequency is obvious, and this favorite position is approximately 1/4 of the beam length to the damaged joint. In practice, if the additional virtual mass is too close to the damaged joint, it is not convenient for practical operation. On the other hand, if the distance is too far from the damaged joint, it is hard to reflect the natural frequency changes.

#### 3.2.2. Damage Estimation by Relative Identification Coefficient

Based on the above analysis, virtual structures are constructed by adding virtual masses at 1/4 of the beam length from its two ends (of course, it is possible to add virtual masses at more positions to obtain more information for structural damage identification). In this way, virtual structures with the same number of semi-rigid joints are constructed. The advantage of this method is that the natural frequency of each virtual structure can reflect, to some extent, the damage possibility of the joints near the additional mass. The difference between the frequencies of the undamaged and damaged virtual structures are calculated, normalized, and defined as the relative identification coefficient of joints.

**Figure 8:**
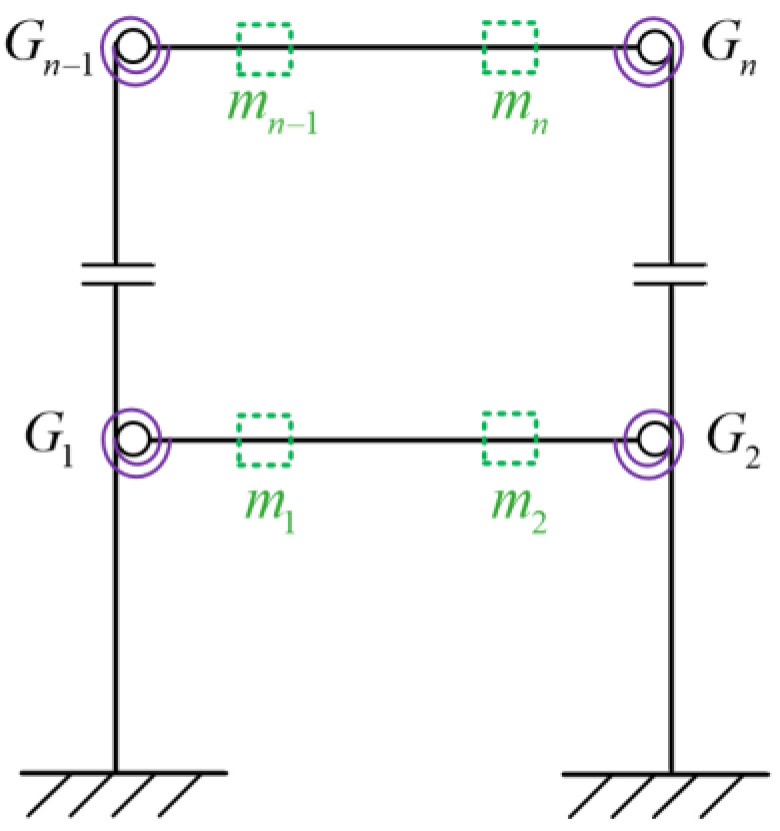
The positions of the added virtual masses.

The positions of the added virtual masses are shown in Figure 8, and the normalization is performed as shown in Equation (6). Assume that there are *n* virtual structures (corresponding to *n* semi-rigid joints), where *C_i_* denotes the relative identification coefficient of the *i*th virtual structure, $\omega_{A,i}$ denotes the natural frequency of a specific order of the *i*th virtual structure obtained from the measured acceleration data of the actual damaged structure, and ${\bar{\omega }}_{i}$ denotes the corresponding natural frequencies of the undamaged virtual structures. It is worth noting that the sensitivity analysis is needed to determine the specific frequency order and the value of additional mass. The greater the *C_i_*, the bigger probability of joint damage.

(6) $$\{\begin{array}{l} \;C_{i}=\frac{\Delta_{i}}{\Delta_{max}}\;\;\;\left(i=1,2,\ldots ,n\right)\\ {\;\Delta }_{i}=\left|\omega_{A,i}-{\bar{\omega }}_{i}\right|\\ {\;\Delta }_{max}=max\left({\;\Delta }_{1},{\;\Delta }_{2},\ldots ,{\;\Delta }_{n}\right) \end{array}$$

### 3.3. Estimation of Damaged Joints Based on Natural Frequency

Although the method described in the above section can quickly and qualitatively identify the potential damage, it cannot accurately determine the location and extent of the damage. Furthermore, a natural frequency-based optimization method is proposed here to obtain more accurate information about joint damage.

#### 3.3.1. The Objective Function Based on Natural Frequency

As in Section 3.2.2, the virtual structures are constructed by adding a virtual mass to the beam at a position 1/4 beam length away from each semi-rigid joint, i.e., there are *n* virtual structures in total. Assuming that Q different virtual masses are added, *N* different virtual structures can be constructed (N=Q×n). All the additional virtual masses can be expressed as $m=\left[m^{1},\ldots ,m^{Q}\right]$, and $m^{q}$ (q=1,2,…,Q) denotes the qth mass. According to Equation (4), the structural frequency response at the *i*th position with additional mass *m^q^* can be estimated, and the corresponding natural frequency of the virtual structure $\omega_{A,i}\left(m^{q}\right)$ can be obtained from the measured acceleration data, which is noted as the identified frequency. On the other hand, the virtual structure of the beam element is designed using the finite element method to optimize the damage factor ***μ***. The mass matrix of the *i*th virtual structure is $M_{i}\left(m^{q}\right)$, when the virtual mass *m^q^* is added at the *i*th position of the finite element model (Figure 8), and the stiffness matrix of the finite element model of the ith virtual structure is *K_i_*(***μ***), when the damage factor is assumed to be ***μ***. Given ***μ***, the natural frequencies $\omega_{i}\left(\mu ,m^{q}\right)$ of the ith virtual structure are calculated mainly by using eigenvalue decomposition of *K_i_*(***μ***) and *M_i_*(*m^q^*). Therefore, an objective function *T*(***μ***) in Equation (7) is constructed using the differences of the identified frequency $\omega_{A,i}\left(m^{q}\right)$ and the calculated frequency $\omega_{i}\left(\mu ,m\right)$ with regard to the given damage factor ***μ***. During the optimization, the value of the objective function *T*(***μ***) is calculated repeatedly with regard to the given damage factor ***μ***. When the damage factor ***μ*** matches the real joint damage factor best, the difference between the frequency $\omega_{A,i}\left(m^{q}\right)$ and the frequency $\omega_{i}\left(\mu ,m^{q}\right)$ is minimized. Therefore, the damage factor ***μ*** that minimizes the objective function *T*(***μ***) is the optimal value of the identified damage factor.

(7) $$T\left(\mu \right)=\frac{1}{2}\overset{Q}{\underset{q=1}{\sum }}\overset{n}{\underset{i=1}{\sum }}{\left(\frac{\omega_{A,i}\left(m^{q}\right)-\omega_{i}\left(\mu ,m^{q}\right)}{\omega_{A,i}\left(m^{q}\right)}\right)}^{2}$$

The objective function shown in Equation (7) can be optimized by intelligent algorithm, such as Genetic Algorithm and the Particle Swarm Optimization method. However, the convergence rate of these intelligent algorithms is slow. During the optimization, $\omega_{i}\left(\mu ,m^{q}\right)$ must be re-modeled every time it is calculated, which requires a lot of computational work. In order to improve the computational efficiency, the natural frequencies $\omega_{i}\left(\mu ,m^{q}\right)$ of the virtual structure are written in an approximate linear expression, for which a Taylor expansion is performed at ***μ*_0_** = **1**, as shown in Equation (8). $R_{i}\left(\mu_{0}\right)$ is a row vector composed of the sensitivity of $\omega_{i}\left(\mu ,m^{q}\right)$ to each damage factor. Combining Equations (7) and (8), the objective function Equation (7) can be optimized using Newton’s method.

(8) $$\omega_{i}\left(\mu ,m^{q}\right)=\omega_{i}\left(\mu_{0},m^{q}\right)+{\frac{\partial \omega_{i}\left(\mu ,m^{q}\right)}{\partial \mu }|}_{\mu =\mu_{0}}\left(\mu -\mu_{0}\right)=\omega_{i}\left(\mu_{0},m^{q}\right)+R_{i}\left(\mu_{0}\right)\left(\mu -\mu_{0}\right)$$

#### 3.3.2. Optimization Based on Improved Orthogonal Matching Pursuit (IOMP) Algorithm

In practice, since not all the joints are damaged, there are usually a large number of undamaged joints in the actual structure. If the potential damaged joints could be picked via some a priori condition of damage sparsity, the optimization accuracy could be improved.

Substitute Equation (8) into the objective function (7) and the *l*_0_ norm of the damage factor increment Δ***μ*** = (***μ*** – ***μ*_0_**) is used as the constraint to construct the objective function for the sparse damage identification, as shown in Equation (9). An Orthogonal Matching Pursuit (OMP) algorithm [35] can be used to optimize the objective function to obtain sparse recognition results. However, the OMP algorithm relies on the iterative screening results of the previous step, and the final optimization result is usually a local optimal solution. The iteration stopping criterion is the estimated damage vector sparsity or the termination threshold set in advance, which is empirical in nature. To address this problem, Zhang et al. [36] proposed an improved OMP (IOMP) algorithm to obtain more stable and accurate sparse damage identification results. The objective function used for damage identification derived in Ref [36] is shown in Equation (9).

(9) $$T\left(\mu \right)=\frac{1}{2}\overset{Q}{\underset{q=1}{\sum }}\overset{n}{\underset{i=1}{\sum }}{\left(\frac{\omega_{A,i}\left(m^{q}\right)-\omega_{i}\left(\mu_{0},m^{q}\right)-R_{i}\left(\mu_{0}\right)\left(\mu -\mu_{0}\right)}{\omega_{A,i}\left(m^{q}\right)}\right)}^{2}+{\left\|\Delta \mu \right\|}_{0}$$

where ***R****_i_*(***μ*_0_**) is the row vector composed of the sensitivity of $\omega_{i}\left(\mu ,m^{q}\right)$ for each damage factor, and the $l_{0}$ norm of the damage factor change vector Δ***μ*** = (***μ*** – ***μ***_0_) is used as the sparse constraint term. Assume that the initial sensitivity matrix of the structure is $R_{0}={\left[R_{1}\left(\mu_{0}\right),\ldots ,R_{i}\left(\mu_{0}\right),\ldots ,R_{q}\left(\mu_{0}\right)\right]}^{T}$, the initial value of the localization vector is η=∅, and the remaining vector $\eta^{*}=\left(1,\cdots ,l,\cdots N\right)$. Take the *S*th iteration as an example to illustrate the screening process of damaged semi-rigid joints as follows.

(1) Constructing the dynamic characteristic residual matrix

(10) $$\{\begin{array}{l} R_{S-1}=\left[r_{S-1,1},\cdots ,r_{S-1,l},\cdots r_{S-1,N-S+1}\right]\\ \varepsilon_{S,l}=(I-R_{S-1,l}^{*}\left(R_{S-1,l}^{*}{)}^{+}\right)r_{S-1,l}\Delta \mu_{l}\\ E_{S}=\left[\varepsilon_{S,1},\cdots ,\varepsilon_{S,l},\cdots \varepsilon_{S,N-S+1}\right] \end{array}$$

The calculation process is shown in Equation (10). $\varepsilon_{S-1,l}$ is the residual vector corresponding to the *l*th node in the *S*−1th iteration step, and Δ*μ_l_* is the damage factor change variable vector corresponding to it. $r_{S-1,l}$ is the *l*th column of $R_{S-1}$, $R_{S-1,l}^{*}$ is the low-dimensional matrix after eliminating $r_{S-1,l}$ from $R_{S-1}$, ${\left(R_{S-1,l}^{*}\right)}^{+}$ denotes the pseudo-inverse matrix of $R_{S-1,l}^{*}$, and ***E_S_*** is the residual matrix of the step iteration.

(2) Screening and eliminating undamaged joint

(11) $${\tilde{E}}_{S}=E_{S}-\varepsilon_{S,m}I$$

(12) $$a_{S,i}=arg\underset{l=1,\cdots ,N-S+1}{min}\frac{{\tilde{\varepsilon }}_{S,l}^{T}r_{S-1,l}}{{\|r_{S-1,l}\|}_{2}}$$

$\varepsilon_{S,m}$ is the column vector composed of the mean values of each row of ***E_S_***. Based on the sensitivity correlation criterion (Equation (12)), the screening parameters of each column vector of ${\tilde{E}}_{S}$ are calculated, and the semi-rigid joint corresponding to the smallest parameter is selected as undamaged;

(3) Sorting damage priority and constructed final residual matrix and location vector

(13) $$\{\begin{array}{l} \varepsilon_{final,N-S+1}=\varepsilon_{S,i}\\ E_{final}=\left[\varepsilon_{final,N-S+2},\cdots ,\varepsilon_{final,N}\right]\cup^{\;}\varepsilon_{final,N-S+1}\\ \eta_{n-S+1}=\eta^{*}\left(i\right)=\iota \\ \eta =\left[\eta_{N-S+2},\cdots ,\eta_{n}\right]\cup \eta_{N-S+1}\\ \eta^{*}=∁\eta^{*}\iota \\ R_{S}=\left[r_{S-1,1},\cdots ,r_{S-1,i-1},r_{S-1,i+1}\cdots r_{S-1,N-S+1}]=[r_{S,1},\cdots ,r_{S,i},\cdots r_{S,N-S}\right] \end{array}$$

Repeat the above steps (1)~(3) to achieve the damage priority ranking by iteration. Finally, the approximate L-curve is drawn using the residual vector parametrization of each semi-rigid joint, and the number of damaged joints is estimated by the inflection point position. The damage locations identified by the above iterative screening correspond to elements in ***μ*** that are non-1 and the rest are 1. The specific damage degree of each joint is determined using the objective function, as shown in Equation (7).

### 3.4. Flow Chart

**Figure 9:**
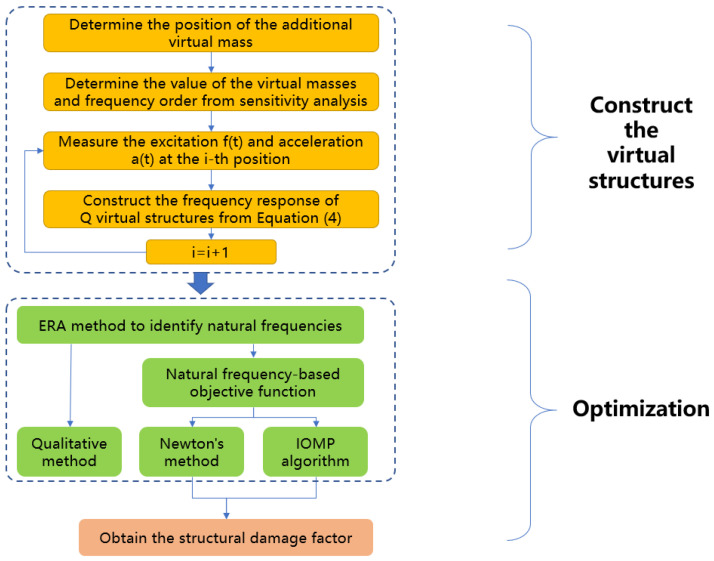
Flow chart of the proposed methods.

To further elaborate the proposed methods, a flow chart is drawn as shown in Figure 9.

## 4. Numerical Simulation of a Frame Structure with Semi-Rigid Joints

**Figure 10:**
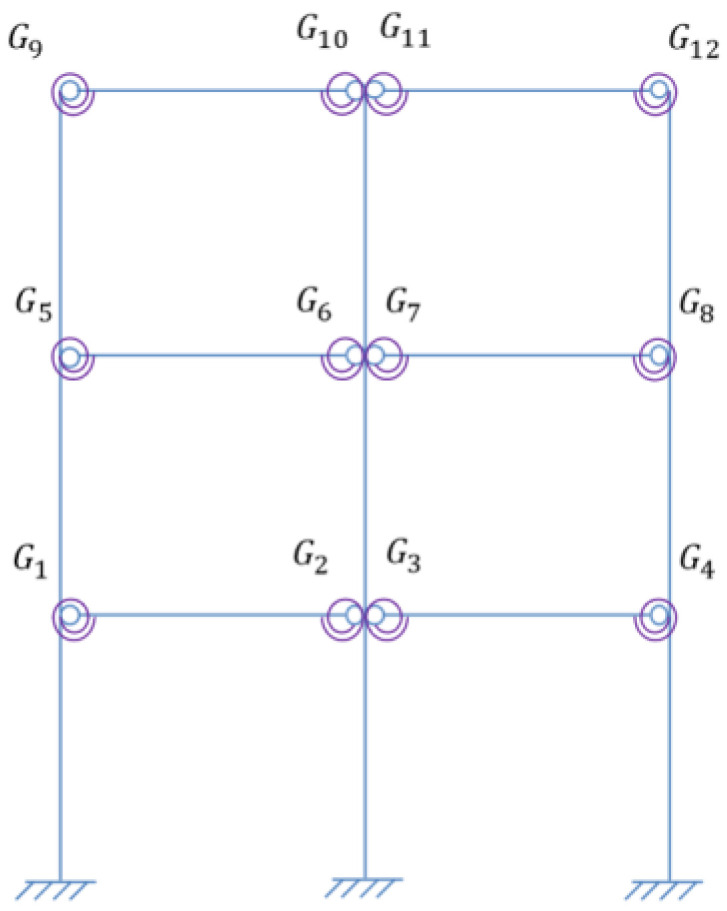
The three-story frame structure model.

In order to verify the proposed methods, damage identification of semi-rigid joints was performed numerically, where a three-story steel frame structure model was adopted, as shown in Figure 10.

### 4.1. Frame Structure Model with Semi-Rigid Joints

#### 4.1.1. Physical Parameters of Frame Structure Model

Assume that the beams and columns have the same cross section size and elastic modulus. The basic parameters of the frame are listed in Table 2. Define the damping of the structure as Rayleigh damping with the damping ratio 0.01 for the first two orders. The beam-column joints $G_{i}\left(i=1,2\ldots 12\right)$ shown in Figure 10 are all semi-rigid and are simulated by a spring element.

#### 4.1.2. Modal Analysis of Frame Structure Model

**Figure 11:**
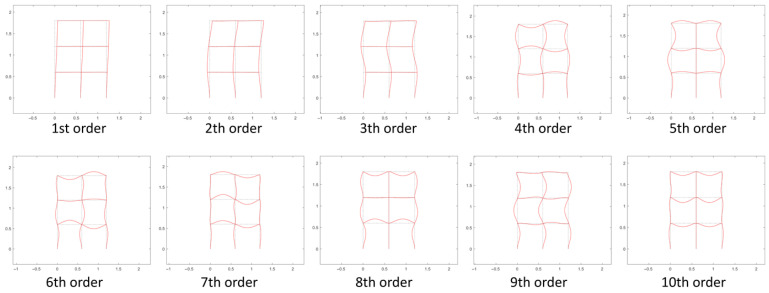
The first 10 orders of modal shapes of the intact structure.

Assume the initial rotational stiffness of the intact joints *R*_0_ = 3150 N · m/rad with *β* = 10, and the first 10 orders of the corresponding structural natural frequencies are shown in Table 3, and the modal shapes are shown in Figure 11.

It can be seen from Figure 11 that the structure mainly performs bending vibrations. The first three orders of modes are dominated by the overall vibration information, and the fourth order and later modes start to show local vibration information. Since the excitation and measurement point is on the beam, the identified original structural modes from the measured acceleration response are likely to be the fourth or more than fourth order. Modes of a higher order are more useful for joint damage identification, but they are not easily excited. Therefore, the specific order of natural frequencies used for damage identification needs to be determined by sensitivity analysis of the virtual structure to joint damage.

**Figure 12:**
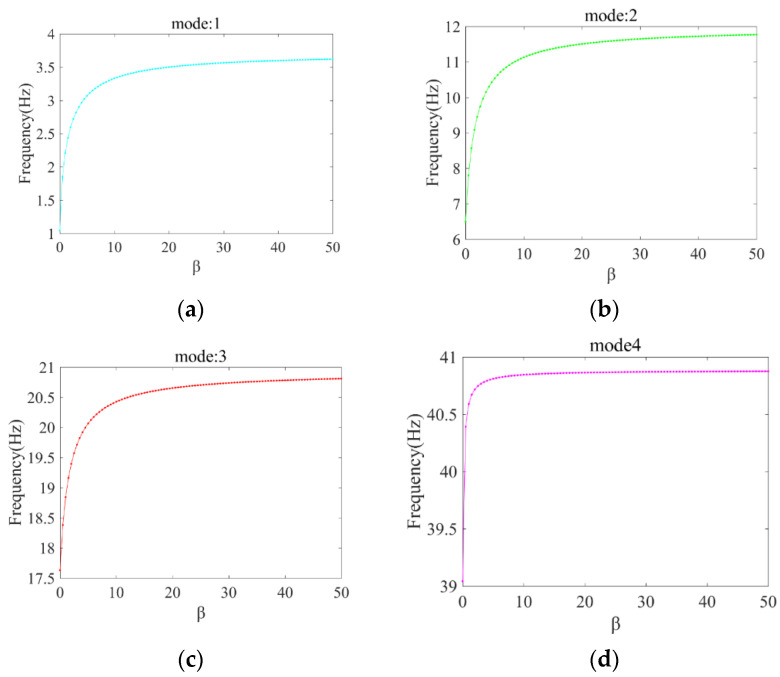
The changes of the first four order natural frequencies with *β*. (**a**) The first order frequency. (**b**) The second order frequency. (**c**) The third order frequency. (**d**) the fourth order frequency.

The changes of the first four orders of natural frequencies with *β* of the frame are calculated respectively and are shown in Figure 12.

As can be seen from Figure 12, the natural frequency is stable when *β* > 25. It means that in this case the change of joints does not influence the dynamic behavior of the structure and so the joints can be treated as a rigid connection at this time. When the value of *β* is between 0.5 and 25, the natural frequency gradually becomes large, which tells that there is an effect of semi-rigid joints on the modal properties of the structure. The above analysis verifies the statement about the definition of the semi-rigid joints referred in Ref. [33] in a certain degree. Therefore, *β* taken as 10 in this study can simulate the performance of semi-rigid joints.

#### 4.1.3. Damage Cases

Two cases of damage conditions are defined as shown in Table 4. In Case 1, only one joint damage is damaged, and four joints are damaged in Case 2. The damage factors are expressed in the vector ***μ*** and need to be identified.

The structure natural frequencies in the two cases are respectively obtained and shown in Table 5, which are compared with the values of the intact structure. It can be seen that in the two cases, the changes of the structural natural frequencies caused by the local joint damage are minimal. This further reflects that the low-order modal information of the structure is not sensitive to local damage and so the damage is difficult to identify, using the estimated structural natural frequencies in this case. By applying virtual masses on the structure to obtain enough useful modal information, the sensitivity of the structural vibration to joint damage can be increased, which is good for increasing the efficiency of damage identification.

### 4.2. Construction of Virtual Structures Based on Adding Virtual Mass Mothed

#### 4.2.1. Potential Positions of the Additional Virtual Mass

**Figure 13:**
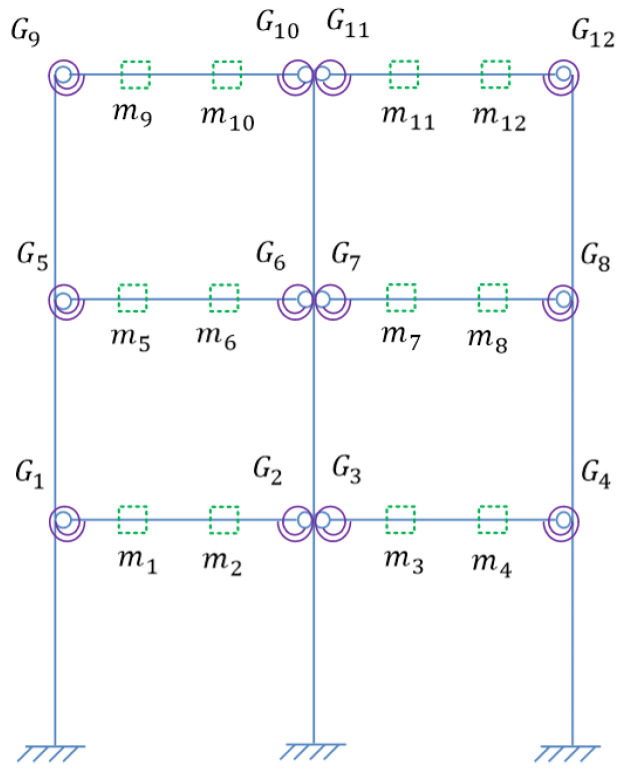
Positions of additional virtual mass.

**Figure 14:**
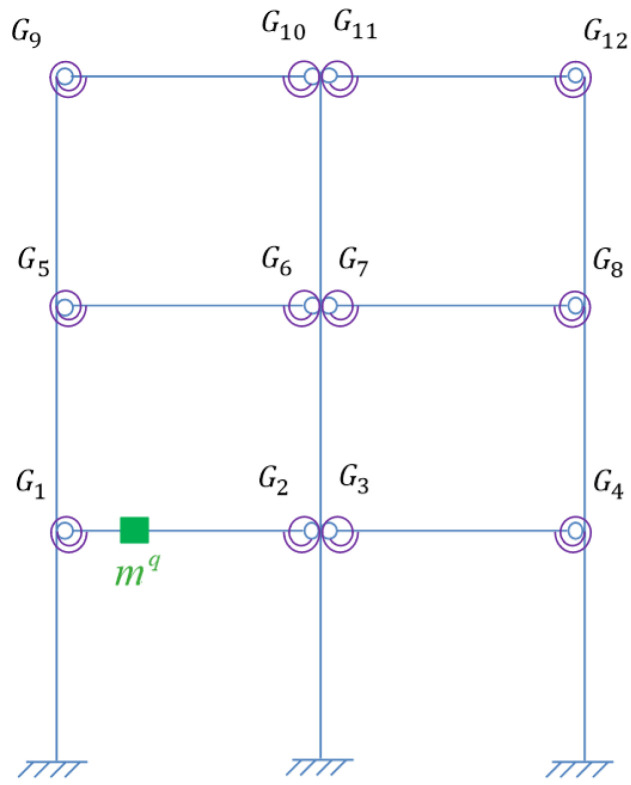
Virtual Structure $S_{1}^{q}$.

Assume one virtual mass added on the damaged frame, the frequency responses of the virtual structure with additional virtual mass can be obtained from Equation (4). According to Section 3.2.1, the specific virtual mass *m^q^* is added near each joint, that is, at the position of 1/4 beam length from the joints. Therefore, there are 12 positions for adding the virtual mass near different joints $G_{i}\left(i=1,2\ldots ,12\right)$, and it turned out to be 12 virtual structures, denoted as $S_{i}^{q}\left(i=1,2\ldots ,12\right)$. The position *m_i_* (i=1,2…,12) where the virtual mass needs to be added is shown in Figure 13, and the virtual structure $S_{1}^{q}$ is shown in Figure 14, which represents the *q*th virtual mass applied at the position *m*_1_.

#### 4.2.2. Determination of the Virtual Mass Value Based on the Sensitivity Analysis

From Equation (5), it shows that the sensitivity of the virtual structure to joint damage changes as the structural parameters change. In order to improve the sensitivity of structural modal information to joint damage, a suitable additional mass value is required to be determined. Firstly, select the virtual mass value from the range between 0 and 7 kg, which is divided into 30 equal parts and the selected value is respectively added to the positions shown in Figure 13. The sensitivity of each virtual structure can be calculated with regard to the change of the virtual mass value.

**Figure 15:**
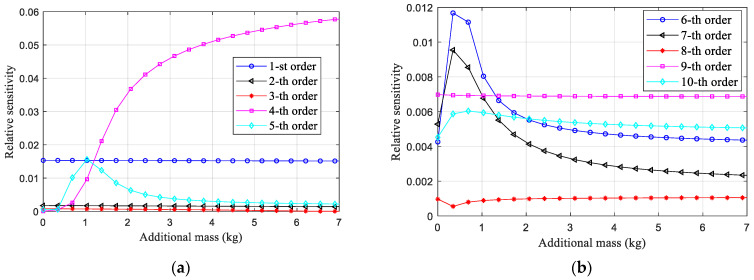
The sensitivity of natural frequency of virtual structure $S_{1}^{q}$ to the damage of joint *G*_1_ regard to different additional mass value. (**a**) From the 1st-order to 5th-order. (**b**) From the 6st-order to 10th-order.

Taking the sensitivity analysis of virtual structure $S_{1}^{q}$ as an example, the sensitivity to the damage of joint *G*_1_ with different value of additional mass *m* is estimated and shown in Figure 15.

From Figure 15, it can be seen that the 4th-order natural frequency of the virtual structure $S_{1}^{q}$ becomes increasingly sensitive to joint damage as the additional mass increases. Although the high sensitivity is good for detecting damage, the influence of the noise would also be enlarged [37]. The sensitivity of the virtual structure increases significantly when the virtual mass is 2–4 kg, so a total of 5 mass values (*Q* = 5) are selected in this interval, denoted as m=[2, 2.5, 3, 3.5, 4], and the qth mass value is denoted by *m*^1^. The virtual masses of the other positions are determined in the same way and can be confirmed to be the same value as the masses at position *m*_1_. Therefore, the virtual mass at each position is denoted by ***m***.

**Figure 16:**
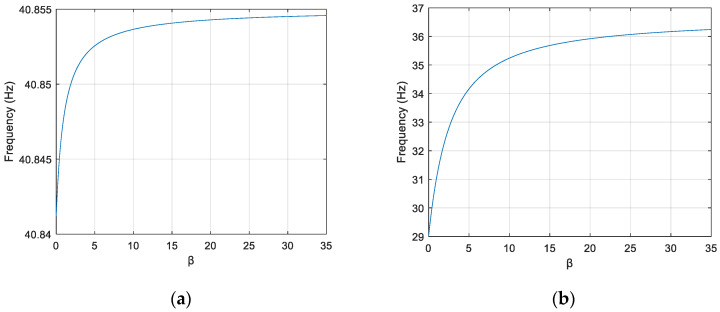
Variation curves of the 4th-order natural frequencies of the original structure and virtual structure $S_{1}^{q}$ with the change of *β* at the *G*_1_ joint. (**a**) Original structure. (**b**) Virtual Structure $S_{1}^{1}$.

When the virtual mass *m*^1^ (2 kg) is added at position *m*_1_, the virtual structure is denoted as $S_{1}^{1}$. The variation of the 4th-order natural frequencies of the original structure and the virtual structure $S_{1}^{1}$ with the *β* of the joint *G*_1_ are shown in Figure 16a,b, respectively. The natural frequency of the virtual structure exhibits a larger change as the *β* of *G*_1_ is varied in the range of semi-rigid joints (0.5 to 25) compared to the original structure. In particular, the slope of the curve is larger when *β* is smaller, indicating a greater change in the 4th-order natural frequency when there is the same degree of damage, which facilitates the identification of joint damage.

#### 4.2.3. Modal Shapes of the Virtual Structure

**Figure 17:**
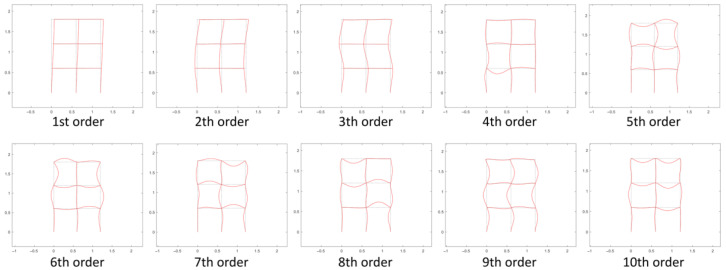
The first 10 orders of modal shape of virtual structure $S_{1}^{1}$.

Take virtual structure $S_{1}^{1}$ as an example to show the modal shapes of the virtual structure. Its modal shapes are obtained using the finite element method as shown in Figure 17. It is observed that for virtual structure $S_{1}^{1}$, the excitation is applied at the position of the added virtual mass *m*^1^, and the vibration of the beam with the added virtual mass is the most pronounced at the 4th-order modal shape. Similarly, for the rest of the virtual structures, the vibration of the beam with the added virtual mass is also the most pronounced at the 4th-order modal shape.

#### 4.2.4. Frequency Response Construction of Virtual Structures with Adding Virtual Mass

**Figure 18:**
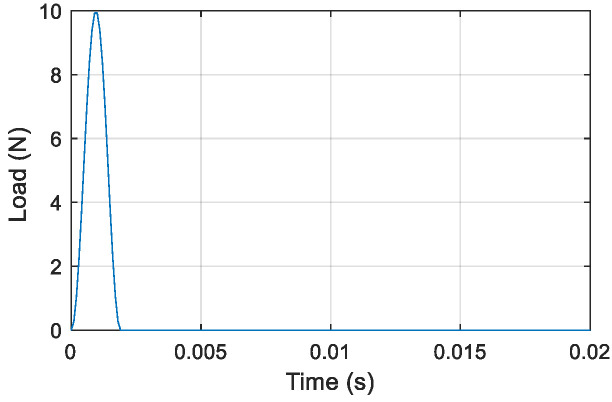
Impulse excitation caused by small hammer.

**Figure 19:**
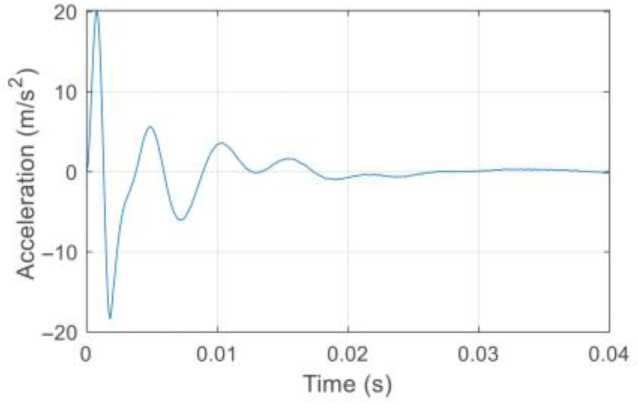
Simulated acceleration response of the actual structure under impulse excitation.

**Figure 20:**
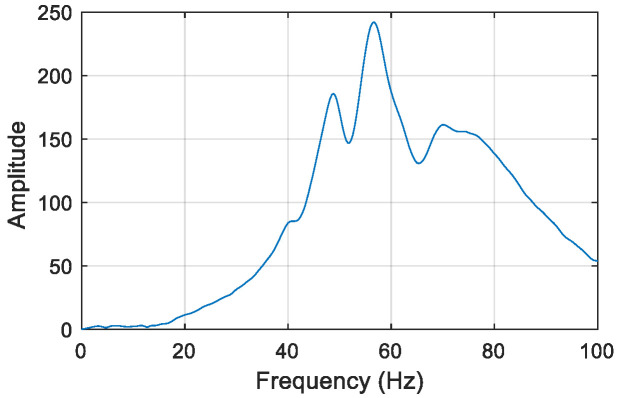
Frequency response of the actual structure.

**Figure 21:**
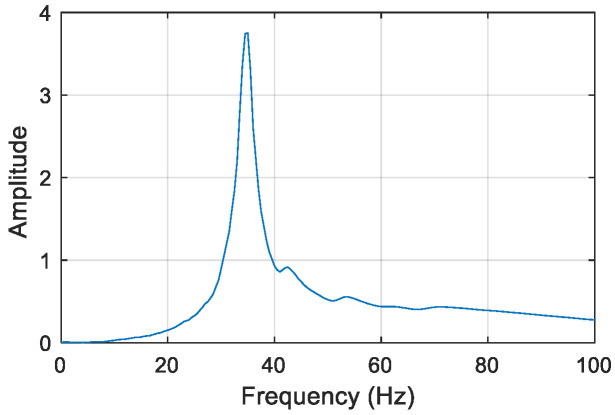
Frequency response of virtual structure $S_{1}^{1}$.

Here, virtual structure $S_{1}^{1}$ is also taken as an example to describe the construction of the frequency responses of the virtual structure. The excitation is applied vertically at position *m*_1_ of virtual mass *m*^1^ of the actual structure, and a sensor is located at the position of *m*^1^ to measure the acceleration response along the direction of the excitation. The impulse excitation is applied using a hammer shown in Figure 18, and the corresponding impulse response of the original structure with 5% Gaussian white noise is simulated and shown in Figure 19. After noise reduction, the frequency response of the damaged structure is obtained by Fourier transform, as shown in Figure 20. Then, the frequency response of virtual structure $S_{1}^{1}$ is derived from Equation (4) as shown in Figure 21, and so the frequency responses of the other virtual structures are obtained.

#### 4.2.5. Natural Frequencies Estimation of the Virtual Structural

It can be seen from Figure 21 that not all the frequencies of virtual structure can be excited to the impulse excitation, and so the specific order of the excited frequency needs to be judged via the finite element model(FEM) of the virtual structure with no damage. Take structure $S_{1}^{1}$ as an example to illustrate this problem. By the FEM of the intact virtual structure $S_{1}^{1}$, the natural frequencies can be obtained and Table 6 lists its first five order natural frequencies. In Figure 21, the frequency corresponding to the maximum of the frequency response is approximately 35 Hz, which is closer to the 4th-order natural frequency listed in Table 6. That’s to say, the modal information obtained of the virtual structure $S_{1}^{1}$ from the measured data is dominated by the 4th order, which also justifies the effectiveness of taking the 4th-order natural frequency as an indicator for damage identification. The analysis process for other virtual structures is similar.

As can be seen from the above, high sensitivity modes are often easily excited out of the mode. Ref. [34] points out the high amplitudes of the frequency response and the high sensitivities of the corresponding natural frequencies are correlated, that is, the larger the amplitude of the frequency response, the higher the sensitivity of the corresponding frequency. According to the frequency response of virtual structure $S_{1}^{1}$ in Figure 21, the amplitude of the fourth mode (35.2 Hz) is the largest, that is, the structure mainly vibrates in the 4th mode. On the other hand, according to the modal shape analysis of applying unit impulse excitation at the position of virtual structure *m*_1_, it is obvious that the 4th mode is more easily excited. The most easily excited modes analyzed by the above two methods are consistent with the most sensitive (4th-order natural frequency) in the sensitivity analysis.

When the frequency response of the virtual structure is constructed, it is theoretically possible to determine the caused natural frequency of the structure by the peak picking method. However, the peak of the frequency response shown in Figure 21 may not exactly correspond to the natural frequency. Therefore, the Eigensystem Realization Algorithm (ERA) [38] is used to calculate the natural frequencies of each virtual structure, which are shown in Table 7. Via ERA, structural modal information can be solved by impulse response. Firstly, the structural impulse responses are discretized in time series, based on which a generalized Hankel matrix is constructed and then by performing singular value decomposition of the Hankel matrix, structural minimum realization matrix is determined. Finally, structural modal parameters are obtained by eigenvalue decomposition of the minimum realization matrix.

The natural frequency accuracy obtained respectively by ERA and the peak picking method for each virtual structure with no damage are compared and shown in Table 7. It shows that although natural frequencies obtained by the two methods are both close to the values estimated by the FEM, the accuracy of ERA is higher.

### 4.3. Qualitative Identification of the Joint Damage Based on Natural Frequencies

For two damage conditions mentioned in Section 4.1.3, the 4th-order natural frequencies of the 12 virtual structures with additional virtual mass of *m*^1^ are respectively estimated by ERA from the measured data, denoted as ‘Estimated’ in Table 8. In order to verify the accuracy of the estimated frequencies of the virtual structure, the frequencies are also estimated using the FEM of the virtual structure with the same damage conditions, denoted as ‘Theoretical’ in Table 8. It can be seen that the estimated values of the frequencies are very close to the theoretical values. This shows that the virtual structural responses are accurately constructed, and the natural frequencies are estimated accurately.

**Figure 22:**
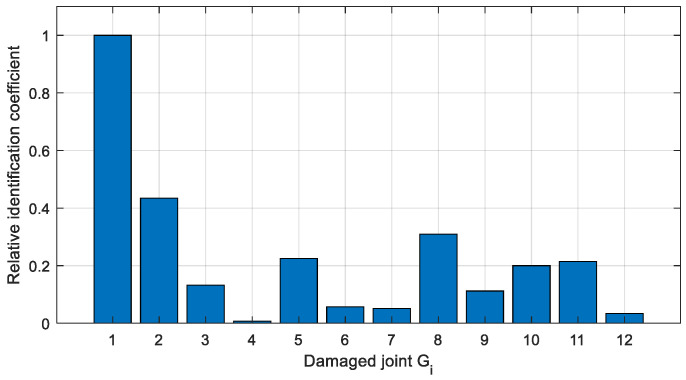
Relative identification coefficient of Case 1.

**Figure 23:**
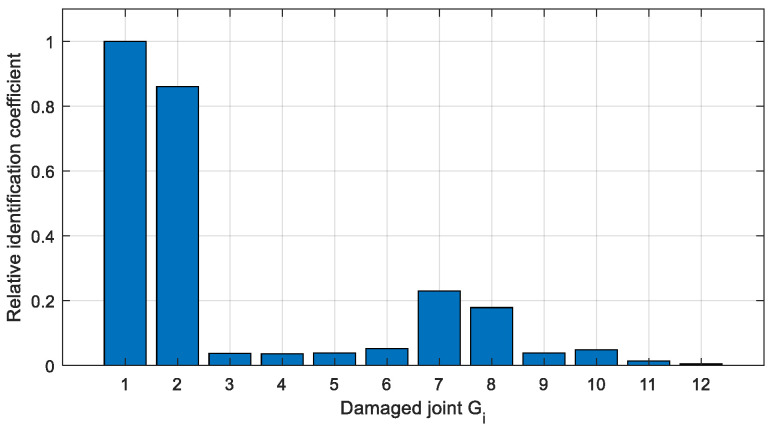
Relative identification coefficient of Case 2.

The relative identification coefficients of the two cases are calculated via Equation (6), as shown in Figure 22 and Figure 23, which can be used to estimate the possibility of different joints damage.

In Case 1 (Figure 22), the relative identification coefficient of joint *G*_1_ is significantly larger than the other joints, and it indicates that this joint has the highest probability of damage, which fits well with that actual damage condition. In Case 2 (Figure 23), the relative recognition coefficients of joints *G*_1_, *G*_2_, *G*_7_, and *G*_8_ are significantly larger than the rest of the joints, and it indicates that the three joints are the most likely to be damaged. However, the damaged joint *G*_6_ is failed to be determined. The relative identification coefficient of joint *G*_8_ is similar to *G*_7_, while joint *G*_8_ is actually intact and so it is misestimated. Therefore, qualitative identification can only identify the damaged joints to a certain extent.

### 4.4. Damage Identification Using Natural Frequency and Frequency Response

#### 4.4.1. Comparison of Identification Results in Low Noise Using Different Methods

**Figure 24:**
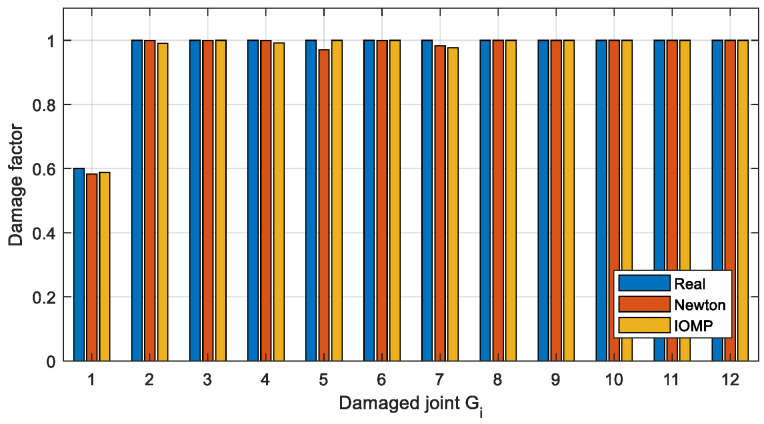
The Estimated damage factors for Case 1.

**Figure 25:**
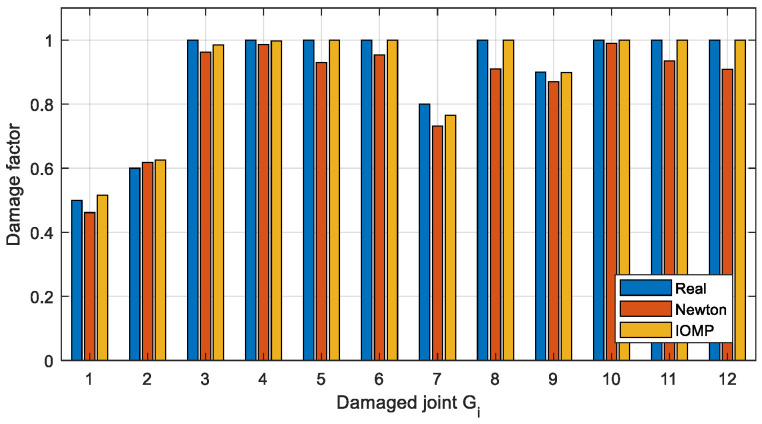
The Estimated damage factors for Case 2.

To get more accurate damage identification results, the finite element model is used to establish the objective function described in Section 3.3. and the damage factors are optimized. Two optimization methods, Newton’s method (Equation (8)) and IOMP algorithm (Equation (9)) are chosen to optimize the natural frequency-based objective function. The damage factors identified by the above three methods are shown in Figure 24 and Figure 25, and the identification errors are calculated using Equation (14), where $\tilde{\mu }$ is the identified damage factor. The identification errors in different damage conditions are shown in Table 9.

(14) $$error_{i}=\frac{\left|{\tilde{\mu }}_{i}-\mu_{i}\right|}{\mu_{i}}\times 100\%$$

It can be seen from Figure 24 and Table 9 that in Case 1 the single joint damage can be identified accurately by the two optimization methods within a 3% error. However, for Case 2 (Figure 25) with multiple joint damage, the identification errors of Newton’s method are obvious, such as the identification results of the undamaged joints *G*_5_, *G*_6_, *G*_8_, *G*_11_, and *G*_12_. In contrast, the IOMP algorithm can effectively improve the accuracy of damage identification, especially to avoid the misestimation of undamaged joints. Therefore, only the optimization method of IOMP is discussed in the later damage identification in different cases.

#### 4.4.2. Influence of High-Level Noise on Identification Results

In practice, the noise pollution on the measurement is inevitable. In order to verify the application of the proposed method in practice, 15% Gaussian white noise is considered in the simulated acceleration response signal. Here, the damage condition in Case 2 is taken as an example. To obtain the natural frequencies of the virtual structures with the high frequency accuracy required by the method, three identical tests were performed and the natural frequencies identified were averaged. When the additional virtual mass is ***m*^1^**, the natural frequency identification results of the first five virtual structures are shown in Table 10. It can be seen that the averaged values of the identified natural frequencies are closer to the actual values compared with the results of the separate test.

**Figure 26:**
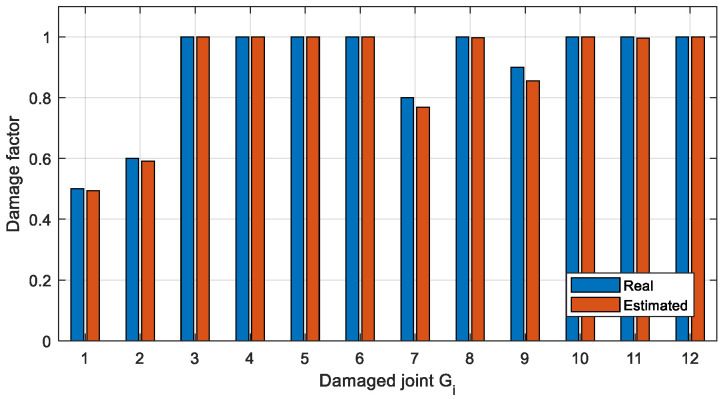
Damage identification results considering high-level noise pollution.

Using the above averaged natural frequencies, the damage factors are identified using the IOMP method and shown in Figure 26. The results of the damage identification in Figure 26 show that the method of attaching different masses to construct more virtual structures and averaging the natural frequencies identified by multiple groups can effectively handle the case of high noise pollution.

### 4.5. Discussion of Other Damage Conditions

#### 4.5.1. The Influence of Beam Damage on Damage Identification

**Figure 27:**
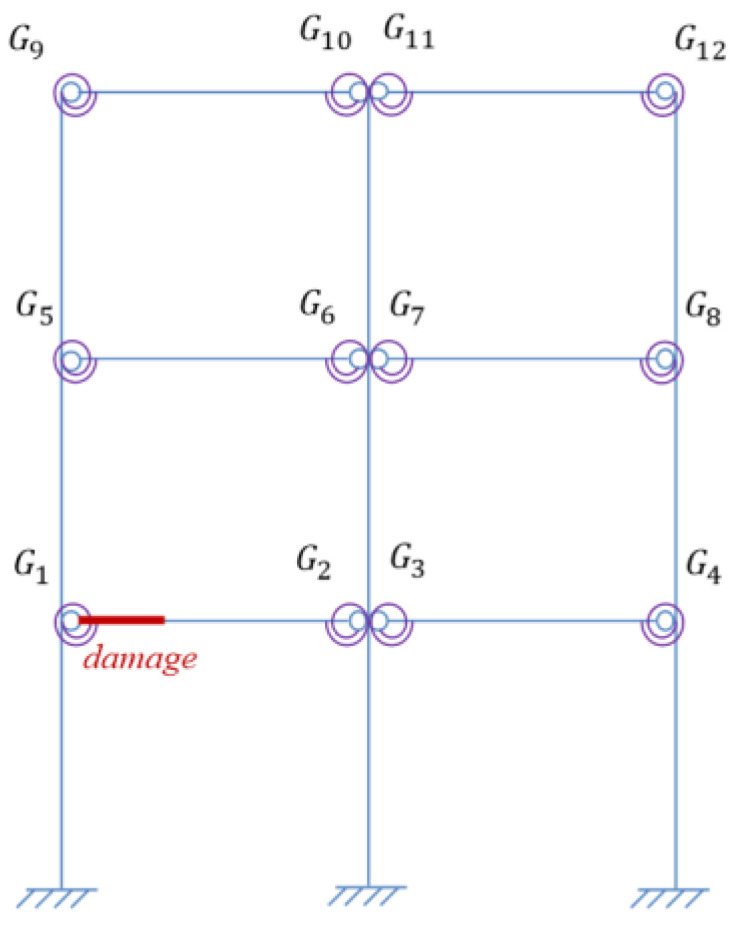
One beam damage near joint *G*_1_.

**Figure 28:**
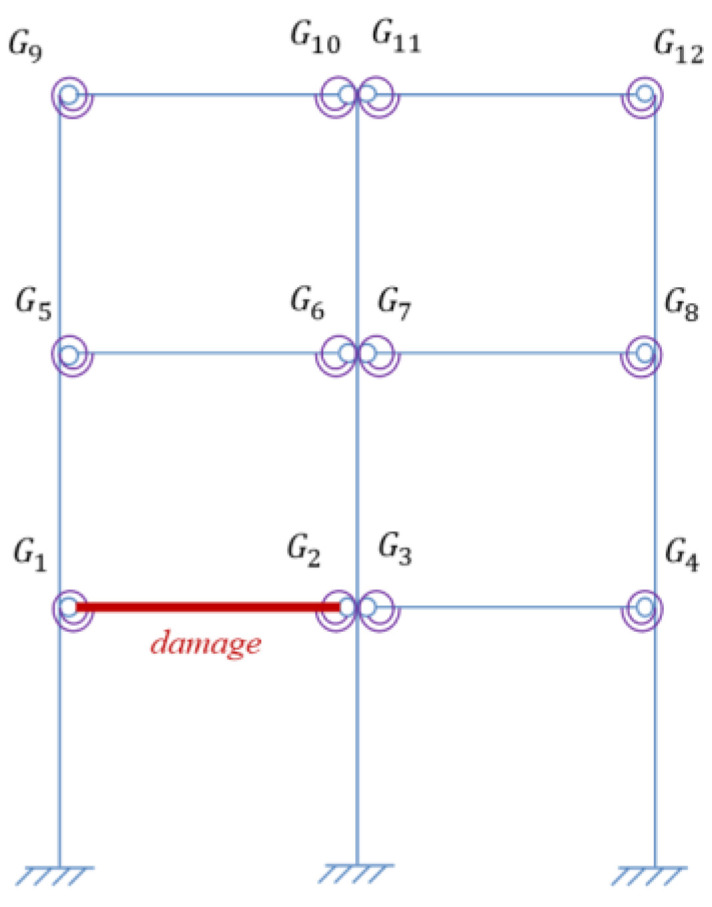
The whole beam damaged.

**Figure 29:**
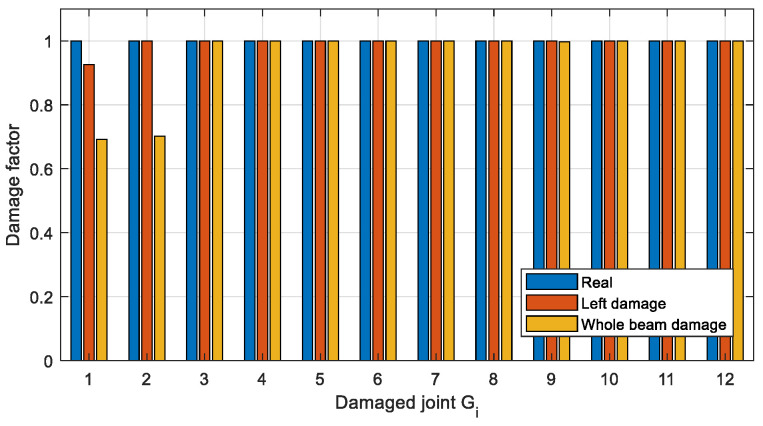
Identification results of joint damages with the existence of different beam damage.

Although semi-rigid joints are more prone to be damaged in practice, beam damage may exist simultaneously with joint damage. To investigate the influence of beam damage on joint damage identification, the beam between joints *G*_1_ and *G*_2_ was assumed to be damaged respectively in the two following cases: one damage near joint *G*_1_ and the whole section damaged. The corresponding damage location is respectively shown in Figure 27 and Figure 28, and the damage factors, i.e., the ratio of the damaged stiffness to the original value, are all set as 0.8. It is assumed that the frame structure for this test has no joint damage and only beam damage. The damage of the joints are shown in the above Case 2. The joint damage is identified by the IOMP method described in Section 4.4, where 5% noise is added. The identified damage factors of the joints are shown and compared in Figure 29.

For the beam damage near joint *G*_1_ labeled ‘left damage’ in Figure 29, the identified damage factor of joint *G*_1_ becomes smaller, i.e., the damage to the beam near the joint is equated to the damage to the joint. When the whole beam is damaged, the identified damage factors of both joints at its ends are drastically reduced. Therefore, when the damage factors of both joints at the ends of the beam are small, it is also possible to equivalently locate the section of the beam that may be damaged.

#### 4.5.2. Effect of the Stiffness Ratio of the Column and Beam on Joint Damage Identification

**Figure 30:**
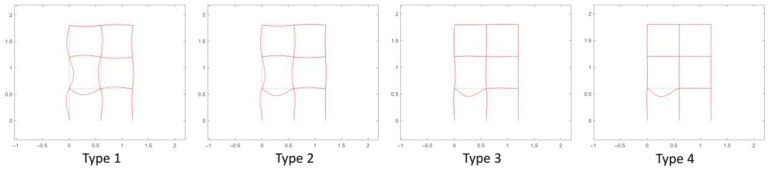
The 4th order modal shapes of virtual structure *S*_1_ under different stiffness types.

**Figure 31:**
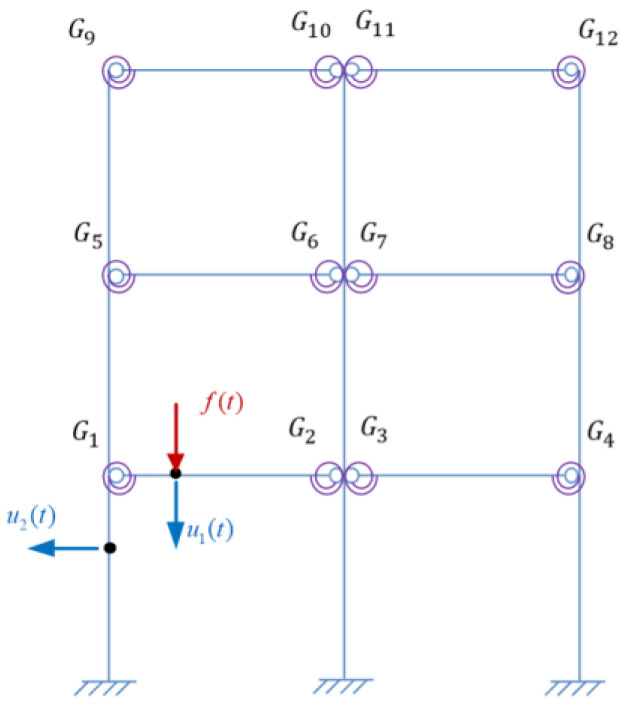
Measurement points *u*_1_ and *u*_2_ set on the structure.

**Figure 32:**
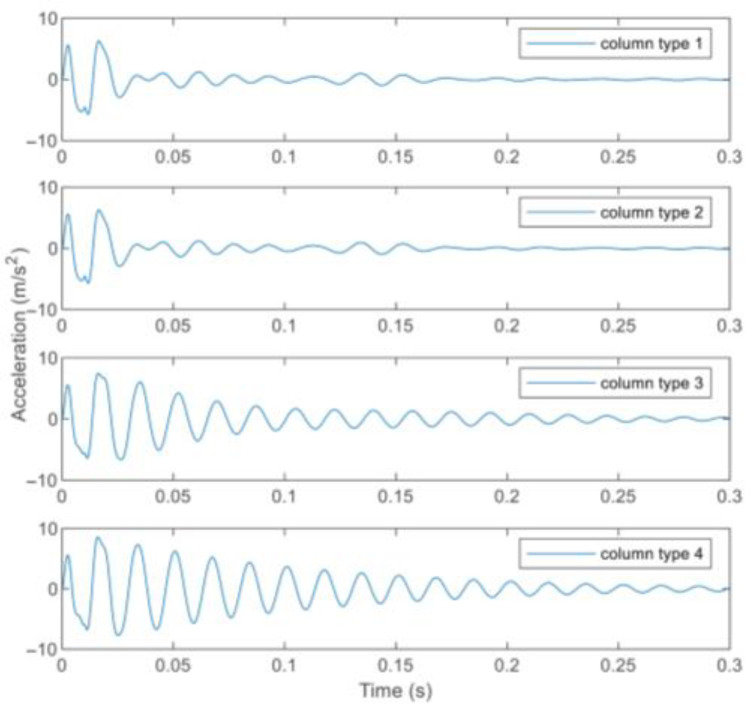
Vertical acceleration response of measured point *u*_1_ on the beam.

**Figure 33:**
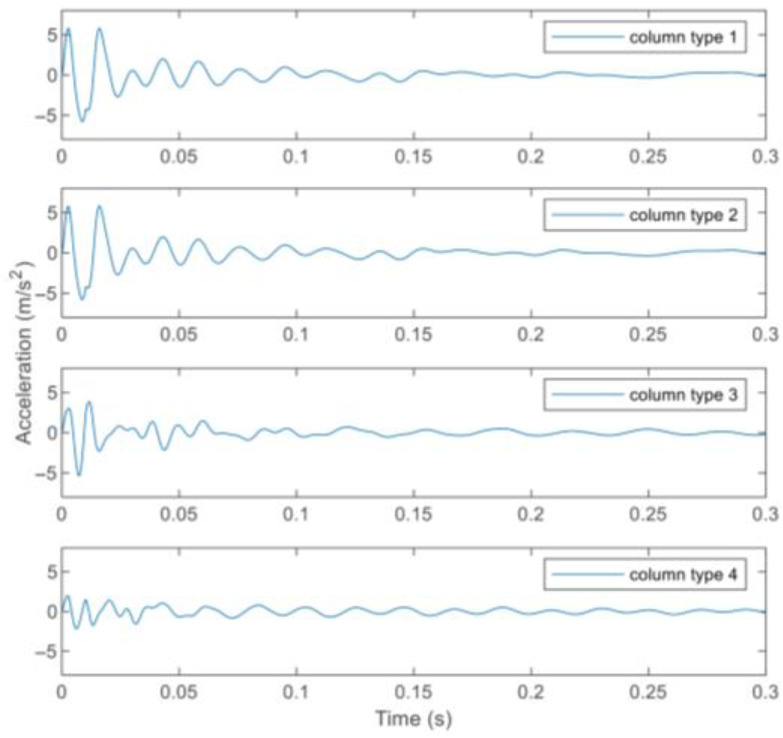
Horizontal acceleration response of measured point *u*_2_ on the column.

**Figure 34:**
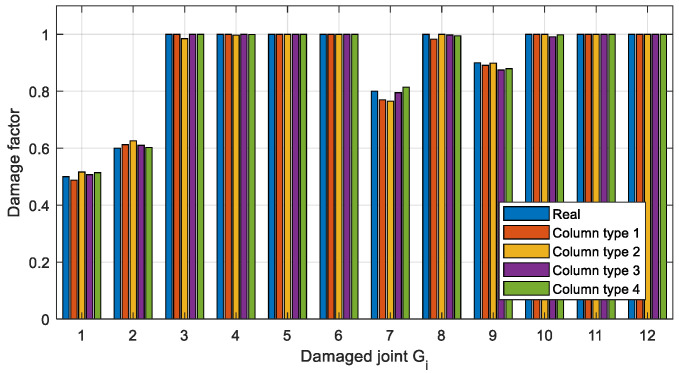
Identification results in case of different stiffnesses ratios.

In this study, the vertical acceleration response of the beam is required for joint damage identification, while the beam vibration may be influenced by the stiffness ratio of the column and beam. Therefore, the influence is investigated by adjusting the stiffness of the column comparatively. The stiffness of the column and the beam calculated from the physical parameters in Table 2 is the same, which is taken as the basic value with the stiffness ratio of the column and beam equaling to 1, and then different stiffness ratios are studied as listed in Table 11. Take the analysis of virtual structure *S*_1_ as an example. With regard to each stiffness ratio, the fourth order modal shape of the virtual structure *S*_1_ is respectively shown in Figure 30. In addition, two measurement points *u*_1_ and *u*_2_ are set on the structure as shown in Figure 31. The measured acceleration responses at *u*_1_ and *u*_2_ in the case of different stiffnesses ratios are respectively shown in Figure 32 and Figure 33. Moreover, regard to each stiffnesses ratio, the identified damage factors using the frequency response-based method with 12 virtual structures are shown in Figure 34, considering 5% noise.

From the modal shapes analysis in Figure 30, it shows that when the stiffness ratio of the column and beam increases, the horizontal vibration of the column becomes smaller and, on the contrary, the vertical vibration of the beam becomes larger, which can also be reflected intuitively by the acceleration responses at *u*_1_ and *u*_2_ in Figure 32 and Figure 33. In other words, the increasing of the beam vibration can more reflect its own characteristics and is more conducive to the identification of joint damage. From Figure 34, it can be seen that the stiffness ratio of the column to beam has little effect on overall joint damage identification.

#### 4.5.3. The Influence of Semi-Rigid Joints with Different Stiffness on Damage Identification

**Figure 35:**
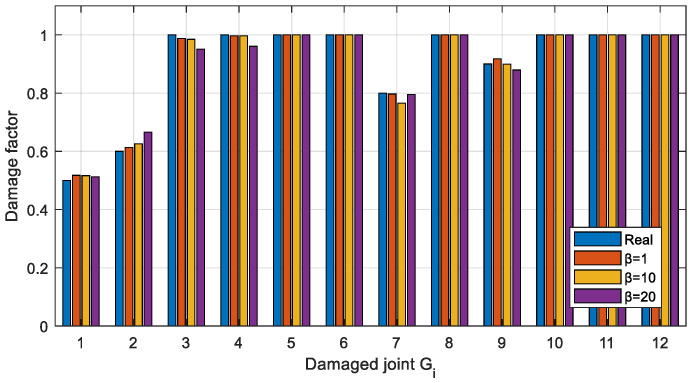
Identification results in case of different *β*.

In order to investigate the effectiveness of the proposed method for structures with different stiffnesses of semi-rigid joints, the results of damage identification for different values of *β* in the structure are shown in Figure 35, where the defined damage condition is shown as Case 2 in Table 4 and the measured acceleration response is obtained with a 5% Gaussian white noise. As can be seen from Figure 35, the proposed method is effective in identifying damaged joints for structures with different *β*. A closer look reveals that the smaller the *β* of the structural joint, for example, joint 2, the higher the damage identification accuracy.

## 5. Conclusions

Aimed at the problem that structural dynamic information is insensitive to local joint damage, this article presents an effective method for semi-rigid joint damage estimation by constructing virtual structures using additional virtual masses. The main conclusions are as follows:

(1) By measuring the excitation and acceleration responses of the actual structure, multiple virtual structures are constructed, which can expand the information of joint damage, and the damage of semi-rigid joints can be effectively identified by the modal information of the virtual structures;

(2) Two natural frequency-based damage identification methods are proposed, the qualitative estimation method and the objective function optimization method. The former can qualitatively estimate the possibility of joint damage but may have misestimation. The latter establishes the objective function based on the natural frequency of the virtual structure and optimizes it using the IOMP method, which can avoid misestimation of undamaged joints and has high accuracy of damage identification even in a high-level noise condition;

(3) When a beam is damaged, the damage is equated to the identified joint. If the beam damage is severe, it will greatly reduce the damage factor of the identified joint, and further analysis of the beam near the joint should be performed at this time.

## Figures and Tables

**Table 1:** Relationship between *β* and joint type.

The Value Range of *β*	Joint Type
*β* < 0.5	articulated joint
0.5 ≤ *β* ≤ 25	semi-rigid joint
*β* > 25	rigid joint

**Table 2:** Basic physical parameters of frame structure.

Physical Parameters	Unit	Value
Story height	m	0.6
Span	m	0.6
Material Density of Beam/column	kg/m^3^	7850
Elasticity modulus of Beam/column	Pa	2.1 × 10^11^
Cross-section width of the beam/column	m	0.05
Cross-section height of the beam/column	m	0.006
Second moment of area	m^4^	9 × 10^−4^

**Table 3:** The first 10 order of natural frequencies of the intact structure (Hz).

Frequency Order	1	2	3	4	5
Frequency value (Hz)	3.33	11.13	20.43	40.85	46.82
**Frequency Order**	**6**	**7**	**8**	**9**	**10**
Frequency value (Hz)	49.62	56.46	56.99	60.14	63.07

**Table 4:** Damage factors of the joints.

Cases	Damaged Joints	Damage Factors
Case 1 (Single damage)	*G*_1_	$\mu ={\left[0.6,\;1,\;1,1,1,1,1,1,1,1,1,1\right]}^{T}$
Case 2 (Multiple damages)	*G*_1_, *G*_2_, *G*_7_, *G*_9_	$\mu ={\left[0.5,\;0.6,\;1,1,1,1,0.8,1,0.9,1,1,1\right]}^{T}$

**Table 5:** The first four order natural frequencies of the intact and damaged structure (Hz).

Damage Cases	The Frequency Order			
	1	2	3	4
Intact	3.33	11.12	20.42	40.85
Case 1	3.30	11.11	20.41	40.85
Case 2	3.26	10.95	20.36	40.83

**Table 6:** The first five order natural frequency of virtual structure $S_{1}^{1}$ with no damage.

Frequency Order	1	2	3	4	5
Frequency value (Hz)	3.33	11.13	20.42	35.24	42.00

**Table 7:** The 4th-order natural frequencies of each virtual structure with additional mass *m*^1^ obtained by different methods in case of no damage (Hz).

Virtual Structure	Real	Peak Extraction Method	ERA Algorithm
*S*_1_	35.24	35.20	35.23
*S*_2_	35.59	35.92	35.79
*S*_3_	35.59	35.48	35.52
*S*_4_	35.24	35.06	35.57
*S*_5_	34.24	33.74	34.23
*S*_6_	34.58	34.67	34.62
*S*_7_	34.58	34.73	34.59
*S*_8_	34.24	34.51	34.51
*S*_9_	32.39	32.25	32.60
*S*_10_	32.93	33.03	33.00
*S*_11_	32.93	33.06	33.06
*S*_12_	32.39	32.51	32.50

**Table 8:** The 4th-order natural frequencies of different virtual structures with additional mass *m*^1^ obtained by the two methods in different damage cases.

Virtual Structure	Undamaged		Case 1		Case 2	
	Theoretical	Estimated	Theoretical	Estimated	Theoretical	Estimated
*S*_1_	35.24	35.23	34.49	34.47	33.70	33.79
*S*_2_	35.59	35.79	35.27	35.25	34.15	34.35
*S*_3_	35.59	35.52	35.57	35.69	35.51	35.54
*S*_4_	35.24	35.57	35.23	35.25	35.20	35.19
*S*_5_	34.24	34.23	34.24	34.41	34.21	34.18
*S*_6_	34.58	34.62	34.58	34.62	34.56	34.50
*S*_7_	34.58	34.59	34.58	34.62	34.29	34.25
*S*_8_	34.24	34.51	34.24	34.48	34.11	33.98
*S*_9_	32.39	32.60	32.39	32.48	32.32	32.33
*S*_10_	32.93	33.00	32.93	33.08	32.89	32.86
*S*_11_	32.93	33.06	32.93	33.10	32.92	32.95
*S*_12_	32.39	32.50	32.39	32.36	32.39	32.38

**Table 9:** Identification errors of damage factor in different cases.

Cases	Optimization Method	Joint Number											
		1	2	3	4	5	6	7	8	9	10	11	12
Case 1	Newton	2.8%	0.1%	0.1%	0.1%	2.9%	0.0%	1.7%	0.0%	0.1%	0.0%	0.0%	0.1%
	IOMP	2.0%	0.9%	0.0%	0.8%	0.0%	0.0%	2.3%	0.0%	0.0%	0.0%	0.0%	0.0%
Case 2	Newton	7.7%	3.0%	3.7%	1.4%	7.1%	4.6%	8.5%	8.9%	3.3%	0.9%	6.4%	9.1%
	IOMP	3.2%	4.3%	1.5%	0.3%	0.1%	0.0%	4.3%	0.1%	0.1%	0.1%	0.1%	0.1%

**Table 10:** The 4th-order natural frequencies of the first five virtual structures with additional virtual mass *m*^1^ (Hz).

Virtual Structure Number	*S*_1_	*S*_2_	*S*_3_	*S*_4_	*S*_5_
Real	33.70	34.15	35.51	35.20	34.22
First test	34.13	35.30	35.28	34.61	34.81
Second test	33.29	34.87	34.30	35.26	33.60
Third test	35.34	35.43	35.19	35.82	34.14
Average of three tests	34.25	35.20	34.92	35.23	34.19

**Table 11:** Different stiffness ratios of the column and beam.

Column Type	Type 1	Type 2	Type 3	Type 4
Stiffness ratios	0.7	1	3	5
